# Building global health research capacity to address research imperatives following the COVID-19 pandemic

**DOI:** 10.1371/journal.pmed.1003753

**Published:** 2021-08-31

**Authors:** Peter H. Kilmarx, Roger I. Glass

**Affiliations:** Fogarty International Center, U.S. National Institutes of Health, Bethesda, Maryland, United States of America

## Abstract

Peter Kilmarx and Roger Glass discuss strengthening health research capabilities as a response to the COVID-19 pandemic.

Research and development of new tools and interventions are necessary to improve global health, as has been made apparent by the Coronavirus Disease 2019 (COVID-19) pandemic [1]. As of mid-July 2021, there have been nearly 190 million cases reported worldwide and more than 4 million deaths; and yet, less than a year after the outbreak was first reported, in an unprecedented global effort, researchers had developed home rapid self-tests [2], established treatment protocols proven effective to improve survival [3], and discovered highly effective vaccines that are already being produced and administered at a large scale [4].

The COVID-19 pandemic also illustrates the importance of having research capacity in place as a critical element of pandemic preparedness. China, with its robust research capacity, was able to rapidly sequence the novel Severe Acute Respiratory Syndrome Coronavirus 2 (SARS-CoV-2) virus in January 2020 [5] and quickly share the results, thereby jumpstarting global development of diagnostic tests and vaccines. In contrast, when outbreaks have occurred in countries with less research capacity, the development of countermeasures—diagnostics, therapeutics, and vaccines—may be delayed. We examined the relationship between a country’s preexisting research capacity and the output of scientific publications in PubMed by the country’s scientists following an outbreak. In the first 2 years after the Ebola outbreak was recognized in Guinea, only 42 papers on Ebola were published with authors with a Guinean affiliation, and there were significant challenges in launching Ebola treatment and vaccine studies. From Brazil, with its strong research infrastructure, 312 publications about Zika were authored by scientists with a Brazilian affiliation in the 2 years after that outbreak was detected, and substantial progress was made in rapidly characterizing the newly recognized, diverse clinical manifestations. Finally, authors affiliated with a Chinese institution published 8,921 articles on COVID-19 since the current outbreak was recognized, with remarkable progress in developing medical countermeasures.

Anticipating significant progress in controlling COVID-19 in 2021, what are the future priorities for global health research? A helpful guide is the 2019 report of the Global Burden of Diseases, Injuries, and Risk Factors Study [6]. This comprehensive synthesis showed that the largest absolute increases in number of disability-adjusted life years between 1990 and 2019 mostly included noncommunicable diseases, i.e., ischemic heart disease, diabetes, stroke, chronic kidney disease, and lung cancer. These illnesses have overlapping risk factors—hypertension, high fasting plasma glucose, high body mass index, tobacco use, and ambient air pollution—which are also highly prevalent and mostly increasing over time [7], further suggesting important areas for research. Notably, some of these diseases and risk factors are also predisposing factors for more severe COVID-19 and prolonged symptoms post-COVID-19.

Many other critical COVID-19 research questions in the NIH-Wide Strategic Plan for COVID-19 Research [8] remain unanswered, and new urgent questions have arisen. These include the following: What can we learn from how genetic and other factors explain the high individual variation in the clinical course of COVID-19 to improve treatment interventions? How can diagnostic tests be optimized for home use and low-resource settings, and can testing platforms be created for rapid adaptation with new emerging pathogens? With the potential for waning immunity and immune escape variants, what strategies will be needed for COVID-19 vaccine booster doses? Lastly, how best can public health interventions and medical countermeasures be delivered to reduce poor outcomes, especially in racial/ethnic minority and other vulnerable populations?

Several other important perspectives on threats to global health cut across multiple disease entities and provide useful frameworks and new imperatives for prioritizing global health research. The One Health concept encompasses interconnections between humans, animals, plants, and the environment and embraces a transdisciplinary approach to address major emerging threats including zoonotic diseases (e.g., COVID-19), vector-borne diseases, antimicrobial resistance, food safety, and environmental contamination [9]. Another framework is Planetary Health, which focuses on the already large and growing health impacts of our extensive disruptions of earth’s systems, especially climate change, but also declining biodiversity, increasing pollution, and shortages of fresh water, land, and ocean resources [10]. In addition, humanitarian crises such as armed conflicts, natural disasters, and disease outbreaks are impacting more people than ever before. New research approaches and partnerships are needed to address evidence gaps and to establish capacities for future challenges [11]. The impact of COVID-19 on routine health services is a striking current example. Lastly, implementation research on promoting the uptake of evidence-based interventions and policies into routine healthcare and public health settings is needed across each of these fields of health research to address persistent gaps between the promise of proven effective innovations and their successful implementation, especially in underserved and marginalized populations that have been more severely impacted by COVID-19 [12].

We believe the greatest priority should be on building health research capacity in low- and middle-income countries (LMICs) where the health burdens and threats are greater and research capacity is often lower than in higher-income countries. Basic pillars of capacity are needed to establish a robust, responsive research environment. Foremost among these is human capacity. Over decades of experience, we have learned that developing research leaders in LMICs requires well-trained individuals with protected time to conduct research and with strong mentorship and networking with both international and local scientists. It is encouraging to see such investigators trained in other research topics such as HIV and tuberculosis now emerge as leaders in their country’s response to the COVID-19 pandemic in Asia, Africa, and the Americas [13]. Other critical capacities include laboratory testing, data management and statistical analysis, clinical trial and community research site development, behavioral and social science, community engagement, ethical review boards, and regulatory systems. A promising emerging approach is to use basic metrics of national and institutional health research capacity to help coordinate and increase efficiency of capacity building efforts, identify and support countries with lowest capacity levels, and facilitate increased research on national health priorities [14]. As we have seen with COVID-19, these capacities can also be rapidly brought to bear to address new health threats. Notably, of the 30 countries taking part in the SOLIDARITY trial of COVID-19 treatment, 16 are LMICs [15]. A critical limitation and emerging priority underscored by COVID-19 is in vaccine research, development, and manufacturing capacity, especially in Africa [16].

COVID-19 has necessitated many other substantial changes in our usual practices of global health research, some of which are likely to persist. Use of digital platforms for telecommunications has exploded. In many settings, telework and distance learning are proving to be very effective and sometimes preferable to the expense and risk of face-to-face meetings. We have seen greater participation in many webinars and network meetings, especially from early-career and LMIC colleagues who did not have the time or the budget for in-person meetings [17]. Importantly, the environmental costs of these virtual meetings are also much lower. Along with increases in telemedicine, there have also been advances in teleresearch whereby participants can be enrolled or followed via their mobile phones, potentially decreasing the costs and barriers to participation and improving study retention. The speed of research, formation of collaborations, and communication of results have all increased remarkably with digital collaboration platforms and rapid publication, including publication of preprints, which are now available on PubMed [18]. International collaboration and coordination in research and regulatory processes have also been critical to the rapid development of medical countermeasures through platforms such as the Access to COVID-19 Tools (ACT) Accelerator of the World Health Organization [19] and the Accelerating COVID-19 Therapeutic Interventions and Vaccines (ACTIV) public–private partnership led by the National Institutes of Health [20].

Unfortunately, there has also been an “infodemic” of misinformation (i.e., any false information) and disinformation (deliberately false or misleading information) around the source and impact of COVID-19 and the science of its prevention and treatment [21]. This is not a new phenomenon, but with the growth of digital platforms with domestic and international rivalries, a major threat has emerged requiring research to better understand and counter that threat.

Finally, COVID-19 is likely to recalibrate perspectives of levels of expertise in north–south relationships among higher- and lower-income countries. At the time of this writing, the public health, healthcare system, and policy approaches in the COVID-19 response of many high-income countries in the north have greatly underperformed in comparison to some lower-income countries in the global south, especially in regard to protecting vulnerable and marginalized populations. This has increased momentum to democratize global health, with the recognition that a new sense of humility and equity will be critical to understand all of the lessons to be learned and improve global health following COVID-19. We applaud the growing role of LMIC scientists in setting the global health research agenda [22].

In conclusion, while the COVID-19 pandemic has already taken a devastating global toll on global health and well-being, it has also provided a strong example of the importance of health research capacity as an essential element of pandemic preparedness. The world faces a wide range of health challenges, from chronic diseases and risk factors to emerging global threats. Building research capacity, especially in countries with lower levels, while learning the lessons of COVID-19, must become a higher priority to achieve our current shared global health goals while increasing resilience to address future health threats.

## Disclaimer

The findings and conclusions in this paper are those of the authors and do not necessarily represent the views of the National Institutes of Health.

## Abbreviations

ACT: Access to COVID-19 Tools
ACTIV: Accelerating COVID-19 Therapeutic Interventions and Vaccines
COVID-19: Coronavirus Disease 2019
LMIC: low- and middle-income country
SARS-CoV-2: Severe Acute Respiratory Syndrome Coronavirus 2
